# Supplementary material for: Polyurethane Foam Rafts Supported In Vitro Cultures of Rindera graeca Roots for Enhanced Production of Rinderol, Potent Proapoptotic Naphthoquinone Compound
Source: Int J Mol Sci. 2021 Dec 21;23(1):56. doi: 10.3390/ijms23010056 (PMC8744616; doi:10.3390/ijms23010056)
Supplement: Supplementary file 1 [file ijms-23-00056-s001.zip › ijms-1450502-supplementary.pdf]

International Journal of
Molecular Sciences

MDPI

Supplementary material

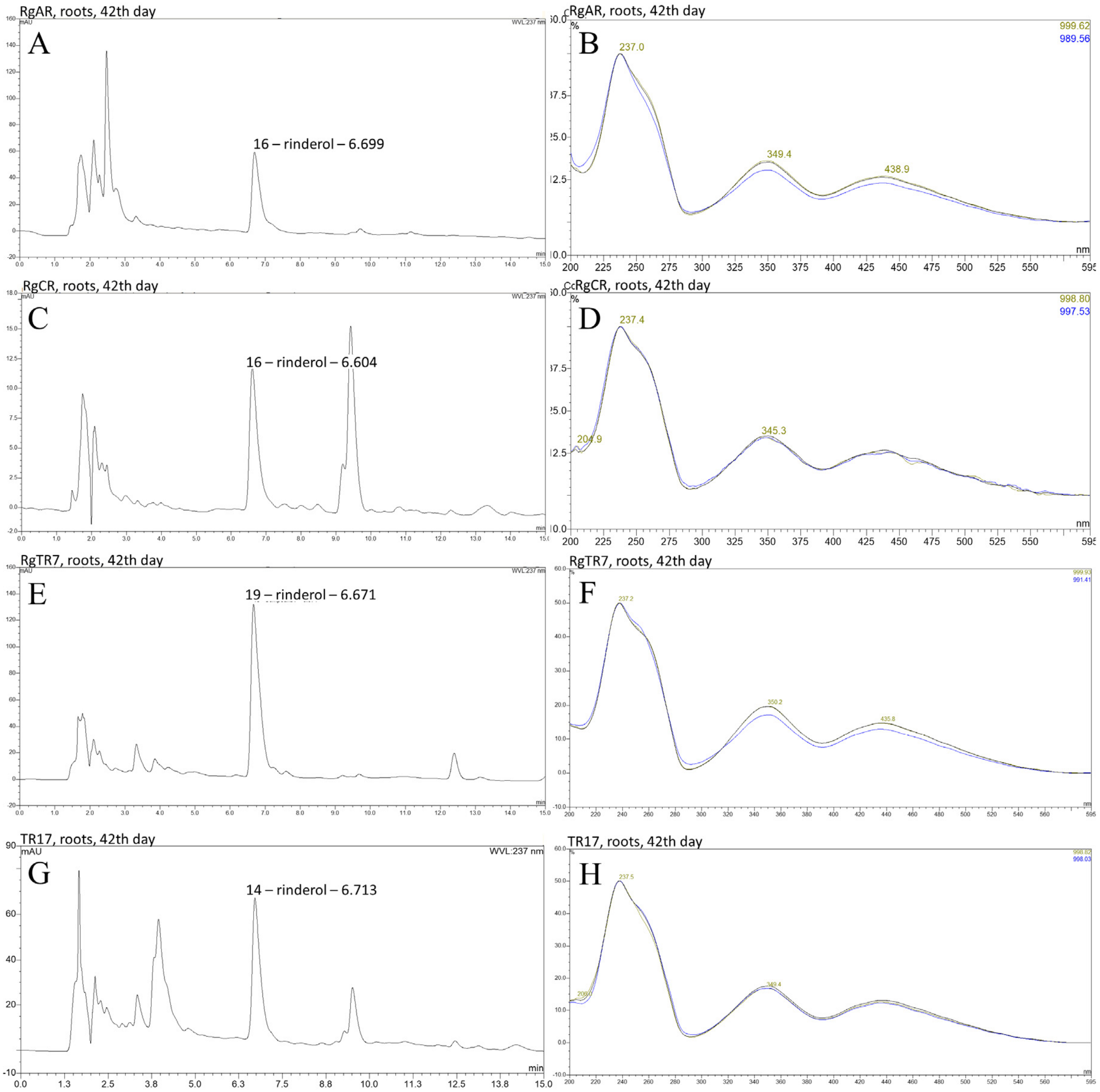

Figure S1. HPLC DAD chromatograms with relative UV-Vis spectrum of extracts obtained from root biomass in control culture systems without immobilization on PUF rafts before rinderol production cessation in untreated roots. Plots demonstrate identification of rinderol in following root lines: RgAR - anatomical roots (A, B); RgCR - regenerated roots (C, D); RgTR7 – transformed root line 7 (E, F), TR17 – transformed root line 17 (G, H). Rinderol specific spectrum used for compound identification λ = 237 nm.

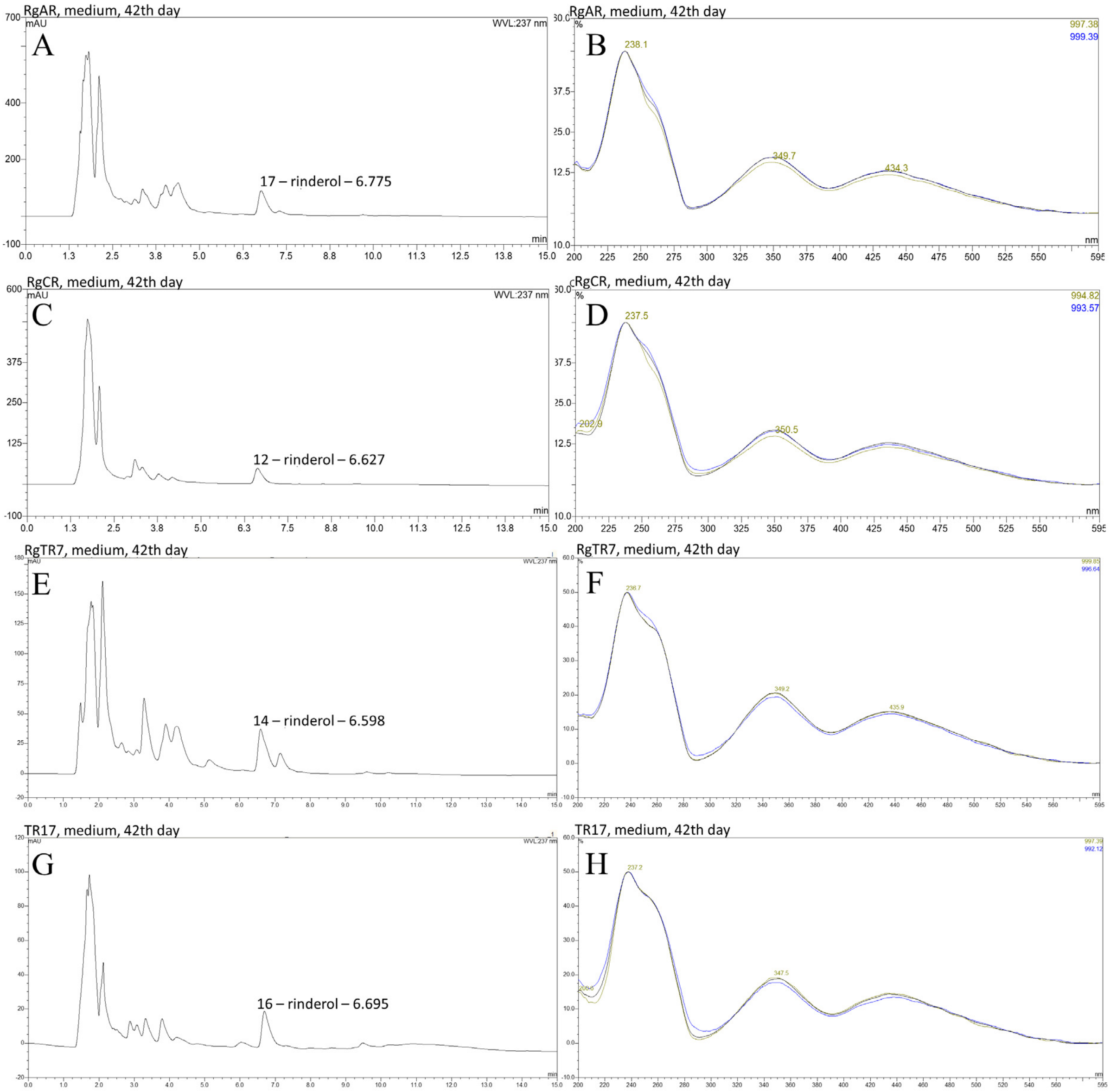

Figure S2. HPLC DAD chromatograms with relative UV-Vis spectrum of extracts obtained from post-culture medium in culture systems immobilized on PUF rafts. Plots demonstrate identification of rinderol in post-culture medium of following root lines: RgAR - anatomical roots (A, B); RgCR - regenerated roots (C, D); RgTR7 – transformed root line 7 (E, F), TR17 – transformed root line 17 (G, H). Rinderol specific spectrum used for compound identification *λ* = 237 nm.

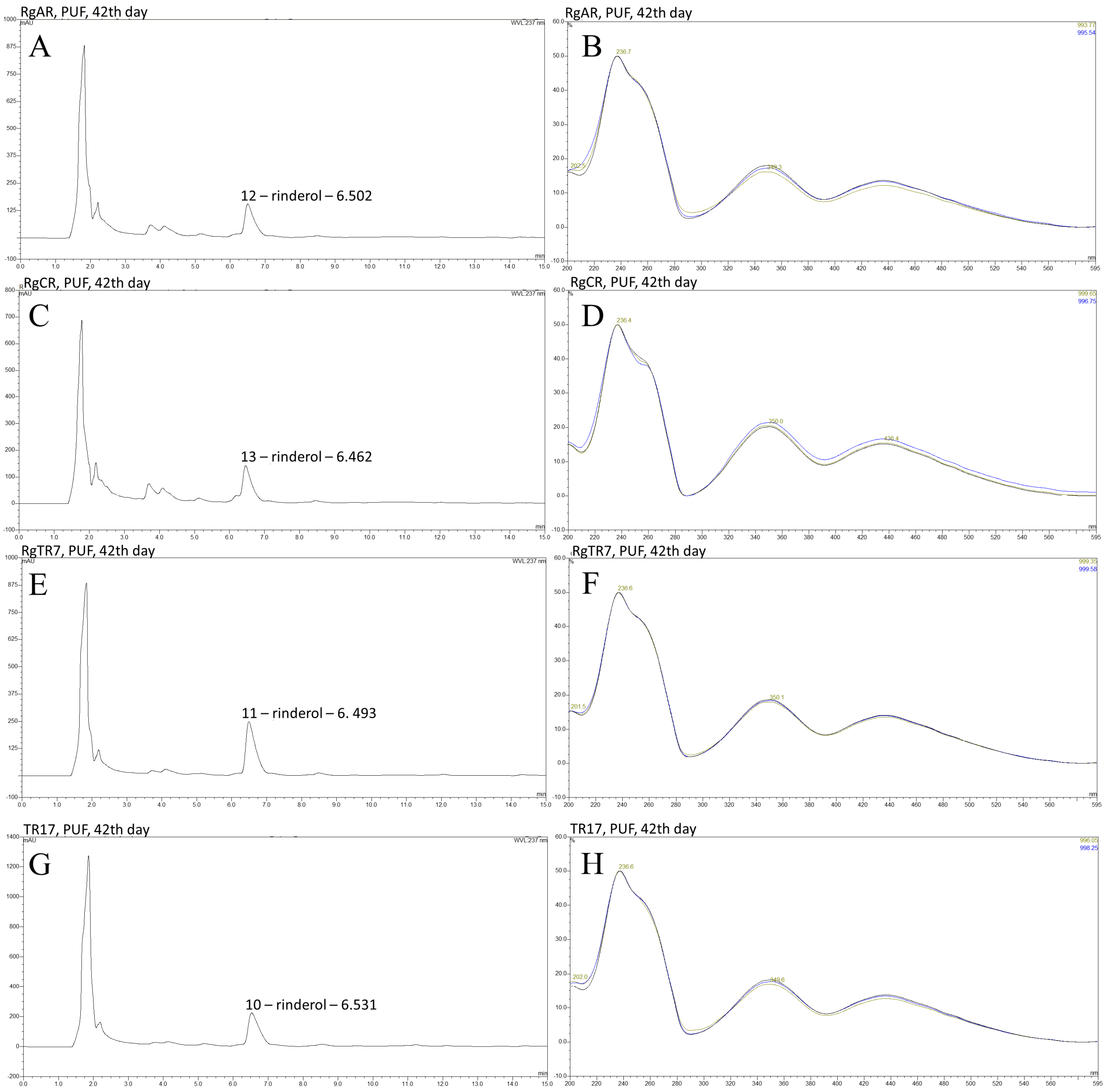

Figure S3. HPLC DAD chromatograms with relative UV-Vis spectrum of extracts obtained from polyurethane foam rafts in culture systems immobilized on PUF rafts. Plots demonstrate identification of rinderol in following root lines: RgAR - anatomical roots (A, B); RgCR - regenerated roots (C, D); RgTR7 – transformed root line 7 (E, F), TR17 – transformed root line 17 (G, H). Rinderol specific spectrum used for compound identification λ = 237 nm.
